# Proteomic Network of Antibiotic-Induced Outer Membrane Vesicles Released by Extensively Drug-Resistant Elizabethkingia anophelis

**DOI:** 10.1128/spectrum.00262-22

**Published:** 2022-07-19

**Authors:** Ming-Hsien Chiang, Fang-Ju Chang, Dinesh Kumar Kesavan, Aparna Vasudevan, Huaxi Xu, Kuo-Lun Lan, Shu-Wei Huang, Hung-Sheng Shang, Yi-Ping Chuang, Ya-Sung Yang, Te-Li Chen

**Affiliations:** a Department of Biology and Anatomy, National Defense Medical Center, Taipei, Taiwan; b School of Materials Science and Engineering, Nanyang Technological University, Singapore, Singapore; c International Genomics Research Centre (IGRC), Jiangsu University, Zhenjiang, China; d Department of Immunology, School of Medicine, Jiangsu University, Zhenjiang, China; e Division of Clinical Pathology, Department of Pathology, Tri-Service General Hospital, National Defense Medical Center, Taipei, Taiwan; f Department of Orthopedic Surgery, Wan Fang Hospital, Taipei Medical University, Taipei, Taiwan; g Department of Microbiology and Immunology, National Defense Medical Center, Taipei, Taiwan; h Graduate Institute of Microbiology and Immunology, National Defense Medical Center, Taipei, Taiwan; i Division of Infectious Diseases and Tropical Medicine, Department of Internal Medicine, Tri-Service General Hospital, National Defense Medical Center, Taipei, Taiwan; j Graduate Institute of Life Sciences, National Defense Medical Center, Taipei, Taiwan; University of Florida

**Keywords:** *Elizabethkingia anophelis*, antibiotic resistance, outer membrane vesicle, proteomics, protein-protein interactions

## Abstract

Elizabethkingia anophelis, a nonfermenting Gram-negative bacterium, causes life-threatening health care-associated infections. E. anophelis harbors multidrug resistance (MDR) genes and is intrinsically resistant to various classes of antibiotics. Outer membrane vesicles (OMVs) are secreted by Gram-negative bacteria and contain materials involved in bacterial survival and pathogenesis. OMVs specialize and tailor their functions by carrying different components to challenging environments and allowing communication with other microorganisms or hosts. In this study, we sought to understand the characteristics of *E. anophelis* OMVs under different antibiotic stress conditions. An extensively drug-resistant clinical isolate, *E. anophelis* C08, was exposed to multiple antibiotics *in vitro*, and its OMVs were characterized using nanoparticle tracking analysis, transmission electron microscopy, and proteomic analysis. Protein functionality analysis showed that the OMVs were predominantly involved in metabolism, survival, defense, and antibiotic resistance processes, such as the Rag/Sus family, the chaperonin GroEL, prenyltransferase, and an HmuY family protein. Additionally, a protein-protein interaction network demonstrated that OMVs from imipenem-treated *E. anophelis* showed significant enrichments in the outer membrane, adenyl nucleotide binding, serine-type peptidase activity, the glycosyl compound metabolic process, and cation binding proteins. Collectively, the OMV proteome expression profile indicates that the role of OMVs is immunologically relevant and related to bacterial survival in antibiotic stress environments rather than representing a resistance point.

**IMPORTANCE** Elizabethkingia anophelis is a bacterium often associated with nosocomial infection. This study demonstrated that imipenem-induced *E. anophelis* outer membrane vesicles (OMVs) are immunologically relevant and crucial for bacterial survival under antibiotic stress conditions rather than being a source of antibiotic resistance. Furthermore, this is the first study to discuss the protein-protein interaction network of the OMVs released by *E. anophelis*, especially under antibiotic stress. Our findings provide important insights into clinical antibiotic stewardship.

## INTRODUCTION

Elizabethkingia anophelis is a Gram-negative, nonfermenting, oxidase-, indole-, and catalase-positive rod-shaped bacterium belonging to the phylum *Bacteroidetes* of the family *Flavobacteriaceae* (1, 2). Some strains of this bacterium have been identified in life-threatening health care-associated infections in fragile, premature babies and immunocompromised individuals, causing bacteremia, lower respiratory tract infection, pneumonia, and neonatal meningitis (3). E. anophelis was first isolated from the midgut of the mosquito Anophelis gambiae in 2011 (4) and caused several outbreaks in South Africa, the United States, Singapore, Hong Kong, and Taiwan (5). Recent investigations into the prevalence and clinical significance of *E. anophelis* have determined that it accounts for a significant proportion of infections, characterized by rapid spread as well as significant morbidity and mortality (30.8 to 70%) (6). Furthermore, clinical isolates of *E. anophelis* exhibited resistance to several antibiotic classes suggested for experimental treatment, including third-generation cephalosporins, β-lactams, aminoglycosides, and carbapenems (7, 8). Due to its innate resistance to most classes of antibiotics, treatment of *E. anophelis* infections poses an insurmountable challenge for clinicians (5). Hence, an analysis of its potential response to different antibiotics is warranted.

Outer membrane vesicles (OMVs) are naturally released from Gram-negative bacteria and serve as potential carriers of loads of relevant molecules for the bacterial life cycle (9), including polysaccharides, nucleic acids, metabolites, and proteins that control biofilm formation (10), quorum sensing (11), antibiotic resistance (12, 13), and/or virulence (14). Various stress conditions can modify the vesiculation of OMVs in response to environmental changes, especially in punitive environments (15). Our recent study demonstrated that most extracellular β-lactamases, such as OXA-58, are selectively secreted via OMVs to confer a carbapenem-sheltering effect on carbapenem-resistant Acinetobacter baumannii (CRAb) (12). The mechanism of OMV biogenesis under stress conditions appears to be different from those of OMV vesiculation and composition. Moreover, bacterium-secreted OMVs enable bacterial communication and host immunomodulatory effects (11). However, the roles of *E. anophelis* OMVs under various antibiotic stress conditions are yet to be demonstrated. In this study, we describe the nanomorphology and cargo content of OMVs released from *E. anophelis* following treatment with antibiotics. Understanding the physical characteristics, protein content, and possible metabolic functions of OMVs secreted by this bacterium will aid in understanding the mechanisms employed by bacteria to deal with environmental stress, counterpart bacteria, or host cells.

## RESULTS

### Antimicrobial susceptibility determined by the MIC.

*E. anophelis* strain C08 was isolated from a blood culture of a 73-year-old female intensive care unit (ICU) patient who suffered from severe bacteremia. The MIC of the strain using the broth microdilution method for 20 antibiotics revealed resistance (R), susceptibility (S), and an intermediate (I) pattern. The strain was resistant to amikacin (MIC > 32 mg/L), aztreonam (MIC > 16 mg/L), piperacillin-tazobactam (MIC ≥ 128 mg/L), ticarcillin-clavulanate (MIC > 128 mg/L), ceftazidime (MIC > 32 mg/L), cefepime (MIC > 32 mg/L), gentamicin (MIC > 8 mg/L), tetracycline (MIC = 128 mg/L), doxycycline (MIC = 4 mg/L), tigecycline (MIC = 8 mg/L), colistin (MIC > 4 mg/L), ciprofloxacin (MIC > 4 mg/L), meropenem (MIC > 8 mg/L), imipenem (MIC > 8 mg/L), chloramphenicol (MIC > 64 mg/L), and trimethoprim-sulfamethoxazole (MIC = 4 mg/L). The intermediate resistance profile was identified for cefotaxime (MIC = 32 mg/L), and the strain was susceptible to doxycycline (MIC = 4 mg/L), minocycline (MIC = 2 mg/L), and levofloxacin (MIC = 2 mg/L) (Table 1). This MIC profiling provides insights into the multidrug resistance (MDR) phenotype of *E. anophelis* strain C08, which is resistant to 13 individual antibiotics and 3 antibiotic-inhibitor combination drugs.

**TABLE 1:** Antimicrobial susceptibility profiles of the *E. anophelis* C08 strain

Antibiotics	Breakpoint (µg/mL)^*a*^			EM361-97^*b*^		C08	
	S	I	R	MIC (µg/mL)	Result	MIC (µg/mL)	Result
Ticarcillin-clavulanate	≤16/2	32/2–64/2	≥128/2	>64/2	I	>128	R
Piperacillin-tazobactam	≤16/4	32/4–64/4	≥128/4	16/4	S	≥128	R
Cefotaxime	≤8	16–32	≥64	ND	ND	32	I
Ceftazidime	≤8	16	≥32	>16	I	>32	R
Cefepime	≤8	16	≥32	32	R	>32	R
Ciprofloxacin	≤1	2	≥4	>2	I	>4	R
Tetracycline	≤4	8	≥16	>8	I	128	R
Doxycycline	≤4	8	≥16	ND	ND	4	S
Minocycline	≤4	8	≥16	<1	S	2	S
Tigecycline	≤2	4	≥8	2	S	8	R
Colistin	≤2		≥4	ND	ND	>4	R
Polymyxin B	≤2		≥4	ND	ND	>4	R
Gentamicin	≤4	8	≥16	>8	R	>8	R
Amikacin	≤16	32	≥64	>32	R	>32	R
Levofloxacin	≤2	4	≥8	>8	R	2	S
Aztreonam	≤8	16	≥32	>16	I	>16	R
Imipenem	≤4	8	≥16	>8	I	>8	R
Meropenem	≤4	8	≥16	>8	I	>8	R
Trimethoprim-Sulfamethoxazole	≤2/38	32/2–64/2	≥4/76	>4/76	R	4	R
Chloramphenicol	≤8	16	≥32	ND	ND	≥64	R

a MIC breakpoints (micrograms per milliliter) applied to isolates according to Clinical and Laboratory Standards Institute (CLSI) “other non-*Enterobacteriaceae*” organisms (there are no interpretive criteria for the susceptibility of *E. anophelis*).

b The MICs of *E. anophelis* EM361-97 were obtained from a previous study by Lin et al. (49). ND, not determined.

### Purification and visualization of OMVs produced by *E. anophelis*.

**FIG 1:**
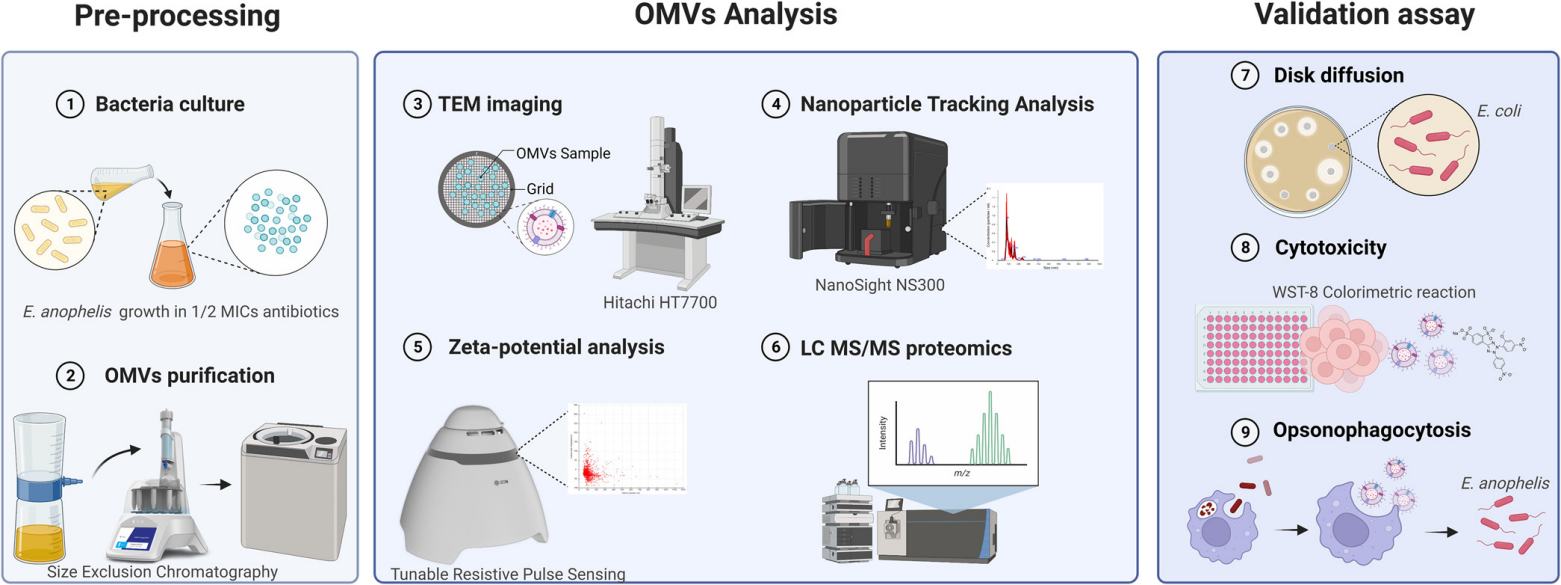
Workflow of *E*. *anophelis* C08 OMV purification and analysis. The experimental strategy uses a three-step approach. The first step involves culturing *E. anophelis* with 1/2 the MICs of antibiotics for 6 h. After ultracentrifugation via a 100K membrane, the supernatant was subjected to size exclusion chromatography (SEC). The OMVs were purified using ultracentrifugation at 150,000 × *g* for 3 h at 4°C. The second step of characterization of *E. anophelis* OMVs was analysis via electron microscopy. Nanoparticle tracking analysis and tunable resistive pulse sensing were used to determine the particle size, concentration, and zeta potential. The OMV component was identified using liquid chromatography-tandem mass spectrometry. After biological pathway and protein-protein interaction analyses, the purified OMVs were subjected to the third step of validation, i.e., a disk diffusion assay for antibiotic sensitivity evaluation, a cytotoxicity assay for virulence activity, and an opsonophagocytosis assay for survival comparison.

**FIG 2:**
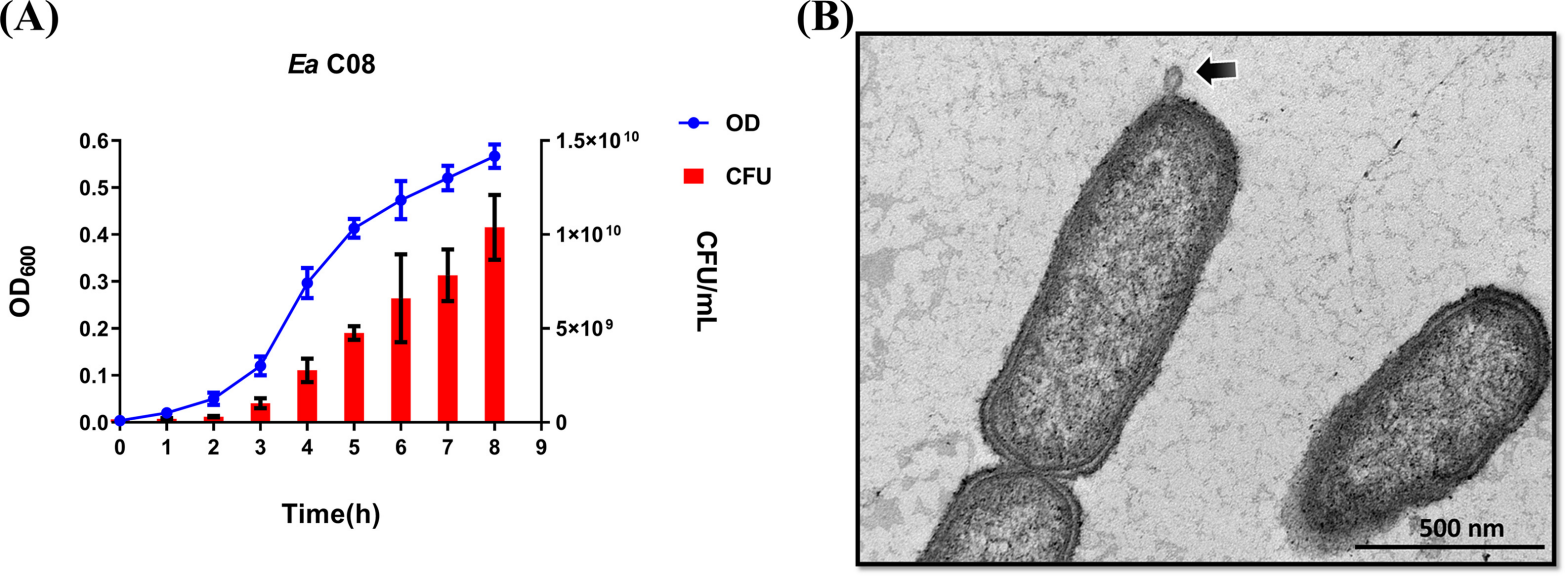
*E*. *anophelis* cell growth and TEM analysis of OMV formation. (A) Growth of *E*. *anophelis* (*Ea*) strain C08 in LB medium. Time points were taken every 1 h for 8 h. Error bars are represented as means ± SD. Data points as shown are the averages from three separate experiments. (B) TEM with heavy metal staining reveals individual OMV vesiculation from mid-log-phase *E. anophelis* cultures. Data are representative of results for five independent OMV preparations.

The OMV purification and analysis strategies are illustrated in Fig. 1. To reduce membrane proteins and cytosolic component contamination caused by cell lysis debris during the stationary phase of bacterial growth, OMV production by *E. anophelis* was measured during log-phase growth (Fig. 2A). Hence, following 6 h of growth, OMVs were harvested for size exclusion chromatography and purification by ultracentrifugation and then characterized and visualized using transmission electron microscopy (TEM). OMVs were observed to bleb out from the outer membrane (Fig. 2B), revealing that *E. anophelis* can produce OMVs via the outer membrane.

### Physical characterization of *E. anophelis* OMVs under different antibiotic stress conditions.

To determine the physical characteristics of *E. anophelis* C08 OMVs following different antibiotic treatments, we collected OMVs after treatment with antibiotics at 1/2 the MIC for 6 h (see Fig. S1 in the supplemental material) and measured the protein quantity, nanoscale size, and particle concentration of OMVs. The purified OMVs were bilayered and spherical by electron microscopy. The results showed that the vesicle sizes ranged from 20 to 300 nm and that *E. anophelis* produced OMVs with an average diameter of ~140 nm (Table 2). Imipenem-induced OMVs (iOMVs) showed the highest OMV particle concentrations. The particle zeta potential also varied, while naturally released OMVs (nOMVs) had an extremely negative charge (−138.9 mV).

**TABLE 2:** Physical characteristics of *E. anophelis* C08 OMVs under different antibiotic conditions

Antibiotic^*a*^	Protein concn (μg/mL)	OMV concn (particles/mL)	CFU/mL	No. of particles/CFU	Mean particle size (nm) ± SD	Mean particle zeta potential (mV) ± SD
Control	10.78	7.66 × 10^9^	7.6 × 10^9^	1.01	117.1 ± 4.5	−138.9 ± 202.5
Amikacin	44.19	8.43 × 10^9^	7.15 × 10^9^	1.18	127.5 ± 2.3	−82.6 ± 48.5
Ampicillin	9.26	5.93 × 10^9^	6.2 × 10^9^	0.96	129.5 ± 7.3	−37.5 ± 33.6
Chloramphenicol	62.41	9.96 × 10^10^	8.6 × 10^8^	115.81	144.0 ± 1.8	−29.1 ± 29.8
Colistin	25.97	3.30 × 10^9^	6.0 × 10^9^	0.55	129.6 ± 1.2	−16.7 ± 18.1
Imipenem	52.09	1.50 × 10^12^	2.12 × 10^10^	70.84	119.5 ± 0.4	−18.4 ± 16.8
Meropenem	22.93	2.18 × 10^10^	5.05 × 10^9^	4.32	139.3 ± 1.9	−9.6 ± 16.5
Minocycline	20.39	1.01 × 10^11^	3.1 × 10^8^	322.58	181.5 ± 0.9	−9.0 ± 58.2
Tigecycline	16.44	6.97 × 10^10^	1.42 × 10^10^	4.91	144.7 ± 0.1	−22.5 ± 17.3

a One-half the MICs were used for the following antibiotics: amikacin (16 μg/mL), ampicillin (32 μg/mL), chloramphenicol (32 μg/mL), colistin (2 μg/mL), imipenem (4 μg/mL), meropenem (4 μg/mL), minocycline (1 μg/mL), and tigecycline (4 μg/mL).

**FIG 3:**
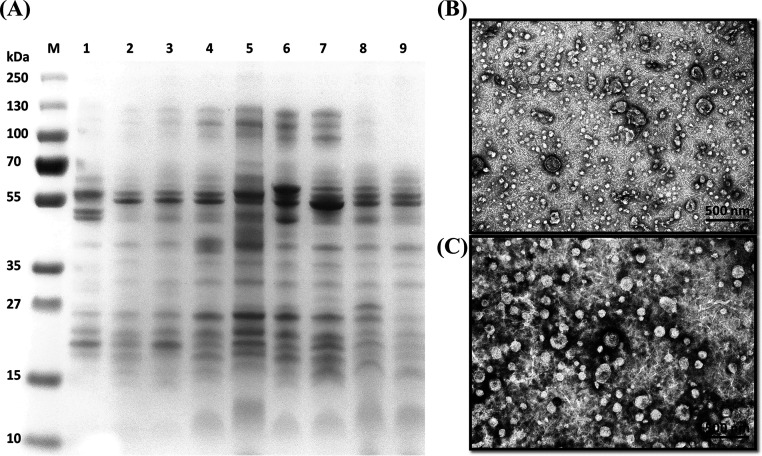
SDS-PAGE and TEM images of OMVs. (A) Coomassie-stained SDS-PAGE gel of the OMVs used in the study. A total of 5 μg of OMV material was loaded into each lane. Lane M, molecular weight markers (in kilodaltons); lane 1, control; lane 2, amikacin; lane 3, ampicillin; lane 4, chloramphenicol; lane 5, colistin; lane 6, imipenem; lane 7, meropenem; lane 8, minocycline; lane 9, tigecycline. (B and C) TEM images of negatively stained nOMVs (B) and iOMVs (C) of *E. anophelis* vesicle samples (0.6 mg/mL). Samples were visualized using a transmission electron microscope operated at 80 kV. Images are representative of results for at least three independent OMV preparations.

The protein distribution pattern of the vesicles was analyzed using SDS-PAGE (Fig. 3A). The OMV preparations showed numerous protein bands in the SDS-PAGE analysis, with a heterogeneous pattern of protein bands among different OMVs. By negative staining, spherical OMVs were seen in the images where single- and double-membrane circular shapes were observed (Fig. 3B), revealing outer-inner membrane vesicles formed by explosive cell lysis. The OMV preparations isolated from cultures in the presence of different antibiotics, including ampicillin, amikacin, chloramphenicol, colistin, meropenem, minocycline, imipenem, polymyxin B, and tigecycline, as captured by electron microscopy, are shown in Fig. S2. Interestingly, the imipenem-induced OMVs exhibited the highest concentration and homogeneity in particle size (Table 2 and Fig. 3C). Although minocycline and chloramphenicol induced the highest numbers of OMV particles/CFU, this might be caused by the lysis of *E. anophelis*.

### Effect of antibiotics on the proteome of OMVs.

**FIG 4:**
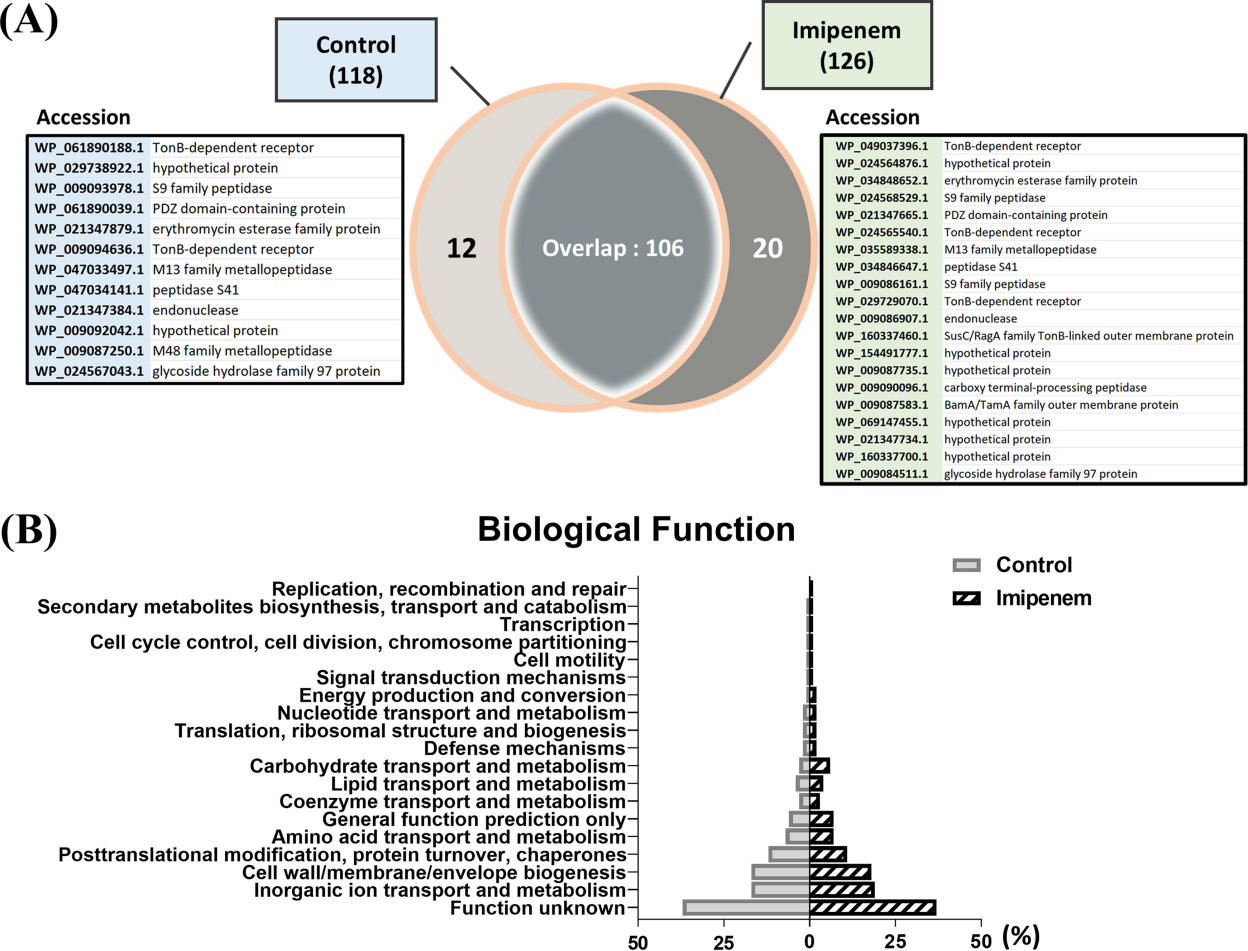
Comparison of OMV proteins derived from imipenem-treated and control strains of *E. anophelis* C08. (A) Venn diagram of differentially expressed proteins according to proteomic analysis. Totals of 118 proteins for nOMVs and 126 proteins for iOMVs were identified, of which 20 proteins were uniquely expressed in the imipenem treatment model. Twelve proteins were unique to the control, and 106 were differentially expressed. (B) Bar graphs categorizing the proteins with 19 differential biological functions, with distributions in percentages.

Proteins from OMVs (antibiotic treated and untreated) were in-gel digested with trypsin and extracted from the gel for proteomic analysis. The complete proteome of extracellular vesicles identified totals of 118 and 126 proteins from the control and imipenem-treated OMV samples, respectively (see the supplemental material). Among these proteins, 106 were identified in both control and treated OMV samples, while 20 and 12 unique proteins were identified in the treated and untreated OMVs, respectively (Fig. 4A). During the proteomics analysis, whole-protein expression was characterized by Gene Ontology (GO) analysis into antibiotic resistance, virulence, and cellular functions (Fig. 4B). Among the identified proteins, the majority were involved in cellular processes; lipid, carbohydrate, nucleotide, and amino acid metabolism; as well as nutrient uptake and transporters for metabolic processes. The most significant change was associated with carbohydrate transport and metabolism (3% in nOMVs and 6% in iOMVs). The specific proteins were an S9 family peptidase, TonB-dependent receptors (TBDRs), and a major facilitator superfamily (MFS) transporter. OMVs play a significant role in pathogenesis, as evidenced by being the source of the second most abundant proteins involved in antibiotic resistance, virulence, and pathogenesis as well as proteins with hypothetical/unknown functionality. This variance was expected considering the strain’s high sensitivity to antibiotic stress and environmental changes, as shown in previous studies (16). As a result, each biological replicate was analyzed separately to determine which proteins were differentially expressed in the control and antibiotic-treated groups.

The localization of OMVs was identified using the Cello2go server. For the control, outer membrane protein functionality was characterized as outer membrane for 51% of proteins, extracellular for 22%, periplasmic for 18%, cytoplasmic for 6%, and inner membrane for 1%. In OMVs from imipenem-treated cells, a similar subcellular localization of proteins was identified (outer membrane for 52% of proteins, extracellular for 24%, periplasmic for 16%, cytoplasmic for 7%, and inner membrane for 1%).

In the present study, we constructed a protein-protein interaction (PPI) network using the String database and analyzed it using ClueGO in Cytoscape software. The following parameters were used to generate a ClueGO network diagram: each node and line represented a term and the correlation between terms, respectively, with a kappa value of 0.4 and a CluePedia gene term cutoff of 50. Furthermore, the color of terms indicates the classification of nodes based on their functions.

**FIG 5:**
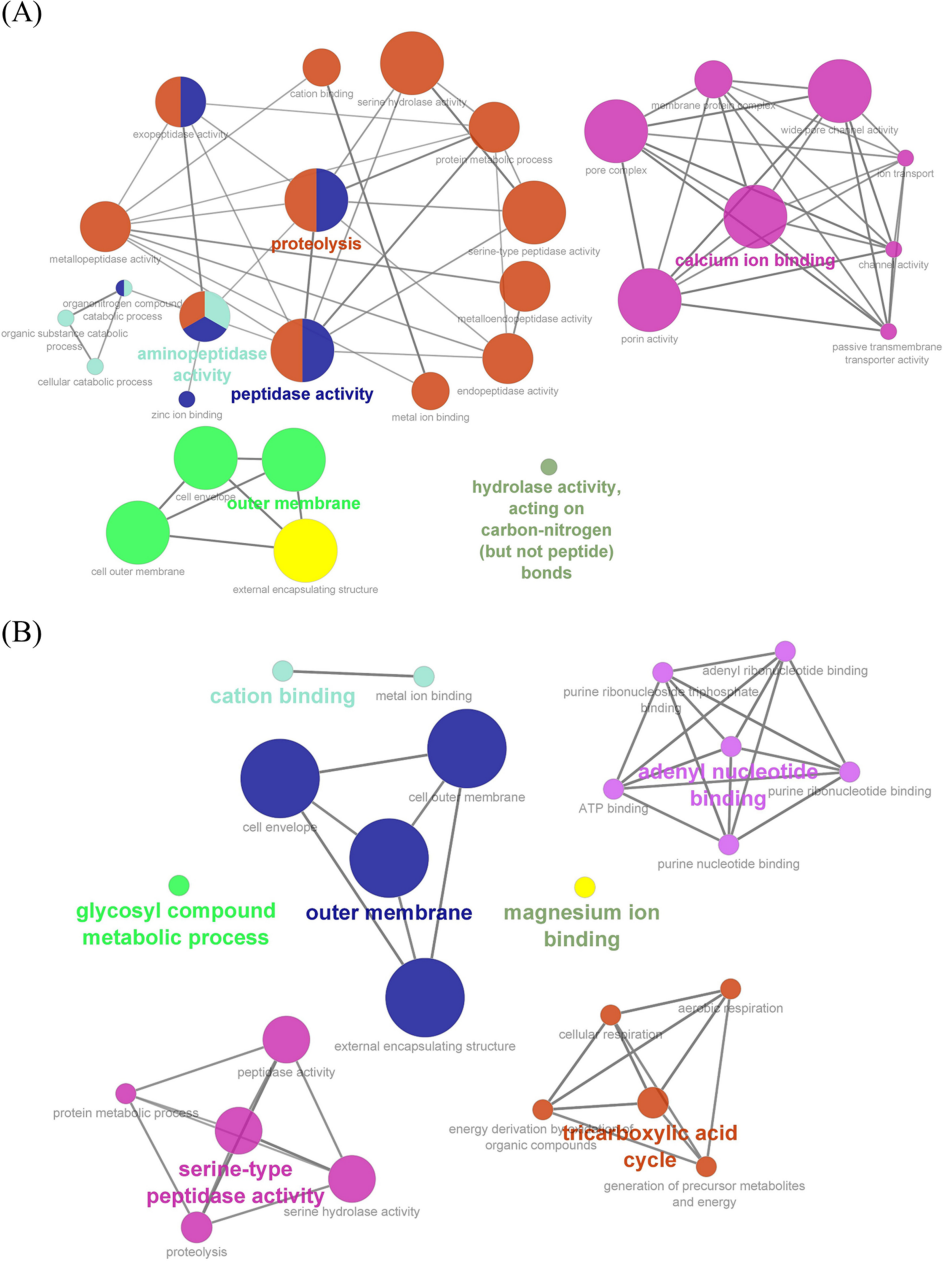
Gene Ontology (GO) enrichment analysis and visualization of genes/proteins from the proteomes of OMVs derived from the control (A) and treated (B) *E. anophelis* C08 strains using the ClueGO/CluePedia plug-in from Cytoscape software. The node colors represent the biological, molecular, and cellular functions of the genes/proteins according to significant associations of related GO terms.

The PPI network results indicated that OMVs from imipenem-treated *E. anophelis* showed significant enrichment in the outer membrane, adenyl nucleotide binding, serine-type peptidase activity, tricarboxylic acid cycle, glycosyl compound metabolic process, magnesium binding, and cation binding proteins (Fig. 5A and B). In OMVs from control strains, most PPIs were associated with proteolysis, calcium ion binding, the outer membrane, and hydrolase activity.

**FIG 6:**
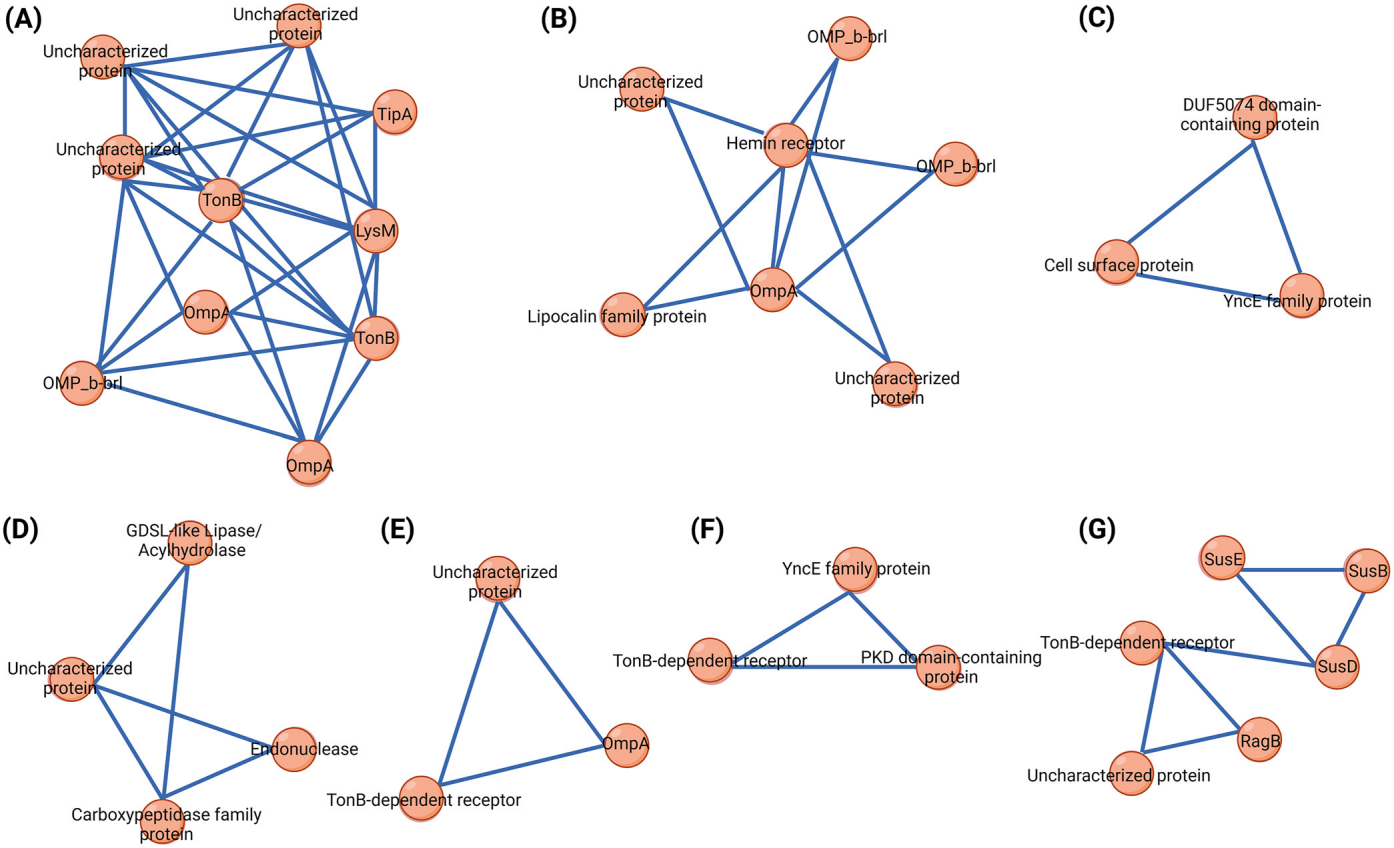
Schematic representation of MCODE clusters of the control (A to C) and treated (D to G) *E. anophelis* C08 strains. Nodes are represented in orange, and the size of the edges is represented using combined scores from the PPI network.

For clustering analysis of interacting proteins, we used the molecular complex detection (MCODE) tool (17). Proteins with more directly connected interactors were grouped based on the number of interactions between nodes. The clustering analysis of the treated and control OMV proteins in the interaction network resulted in four and three densely interconnected clusters, respectively (Fig. 6). The MCODE algorithm includes a cluster and uses each node’s degree (number of direct nodes). MCODE provides a score, which is calculated based on cluster density (Tables 3 and 4). For the treatment group, cluster 4 contained 6 nodes and 7 edges, while clusters 1 to 3 included 4, 3, and 3 nodes and 5, 3, and 3 edges, respectively. In the control group, cluster 1 contained 10 nodes and 33 edges, while clusters 2 and 3 had 7 and 3 nodes and 11 and 3 edges, respectively (Tables 3 and 4).

**TABLE 3:** Clustering analysis of the interacting proteins of *E. anophelis* C08 OMVs of the control using MCODE

Cluster	MCODE score	No. of nodes	No. of edges	Node IDs
1	7.333	10	33	OmpA, OMP_B-BRL_2 domain-containing protein, LysM repeat protein, OmpA family protein, TonB-dependent receptor, uncharacterized protein, putative TonB-dependent receptor, uncharacterized protein, uncharacterized protein, thiol:disulfide interchange protein
2	3.667	7	11	Putative outer membrane protein, OMP_B-BRL_2 domain-containing protein, uncharacterized protein, lipocalin family protein, carboxypeptidase family protein, putative hemin receptor, uncharacterized protein
3	3	3	3	DUF5074 domain-containing protein, YncE family protein, putative cell surface protein

**TABLE 4:** Clustering analysis of the interacting proteins of imipenem-treated *E. anophelis* C08 OMVs using MCODE

Cluster	MCODE score	No. of nodes	No. of edges	Node IDs
1	3.333	4	5	Endonuclease, carboxypeptidase family protein, GDSL-like lipase/acylhydrolase, uncharacterized protein
2	3	3	3	OmpA, TonB-dependent receptor, uncharacterized protein
3	3	3	3	YncE family protein, PKD domain-containing protein, TonB-dependent receptor
4	2.8	6	7	SusB, SusD, SusE, RagB, TonB-dependent receptor, uncharacterized protein

### Preliminary evaluation of results from proteomic analysis.

**FIG 7:**
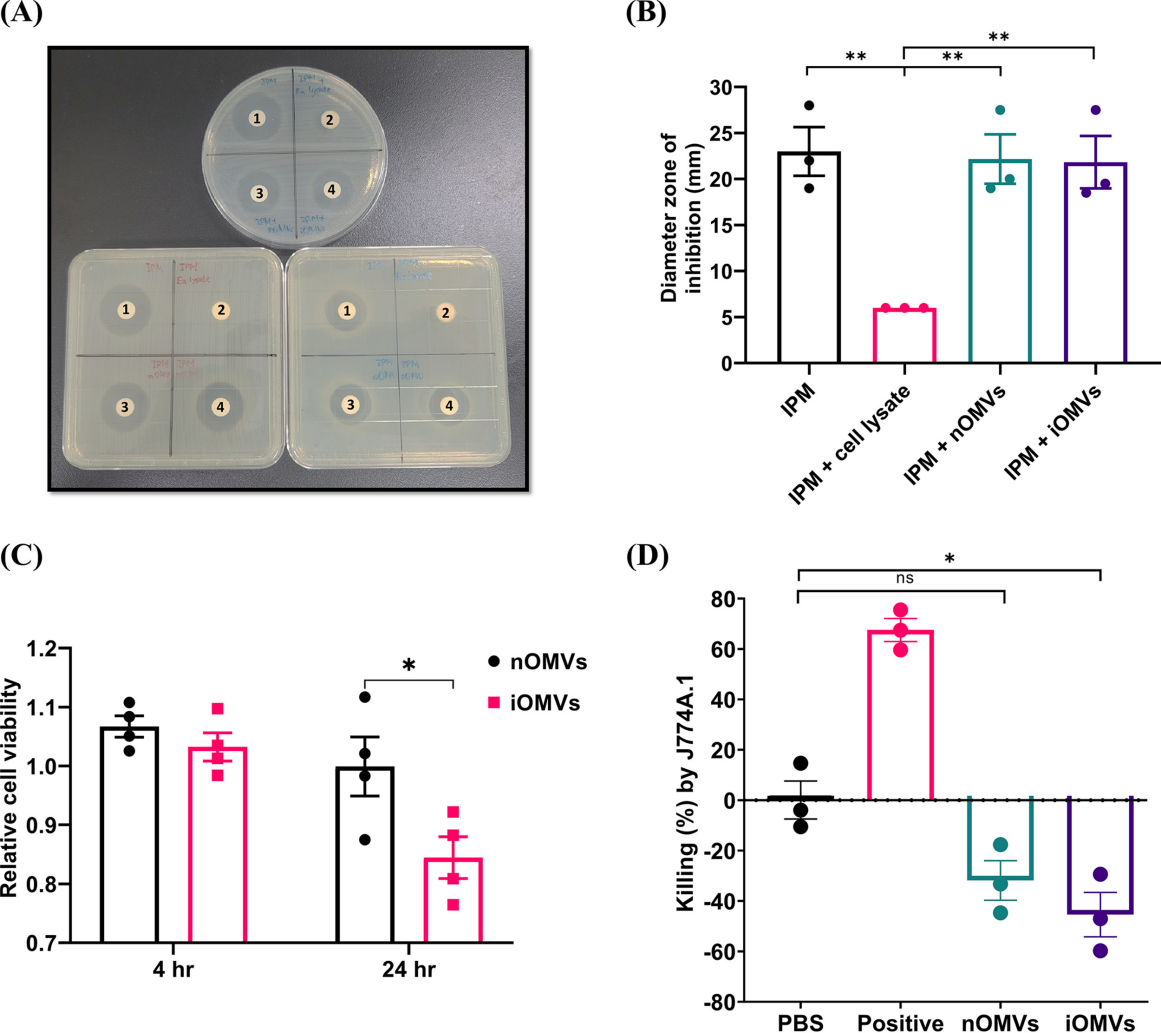
Disk diffusion test of imipenem disks (10 μg) against Escherichia coli ATCC 25922. Disk 1, imipenem (IPM); disk 2, imipenem with the *E. anophelis* cell lysate (10 μg); disk 3, imipenem with nOMVs (10 μg); disk 4, imipenem with iOMVs (10 μg). The inhibition zone was interpreted as positive. (A) Images of the disk diffusion test showing the inhibition zones of E. coli ATCC 25922. (B) Measurement of the diameter of the inhibition zone in panel A. (C) OMV cytotoxicity activities were determined using a lactate dehydrogenase (LDH) release assay in A549 cells treated with 10 μg of OMVs for 4 and 24 h. Relative cell viability was calculated using the ratio of nOMV/iOMV treatment to the PBS control. (D) Opsonophagocytic uptake of bacteria inhibited by OMV treatment. Bacterial killing rates were determined by comparing the number of reduced CFU with those observed using PBS-immunized antiserum. The positive control was hyperimmune serum from OMV-immunized mice (our unpublished data). Data correspond to the results from three independent experiments and are shown as means ± standard errors of the means (SEM). One-way ANOVA with Bonferroni’s multiple-comparison test was used (*n* = 3). *, *P* < 0.05; **, *P* < 0.01; ns, not significant.

We designed three assays to evaluate the proteomic analysis findings *in vitro*. First, for the disk diffusion assay to assess the potential carbapenemase activity of OMVs against imipenem, imipenem disks were placed on each side of a plate, which was inoculated with phosphate-buffered saline (PBS), 10 μg of an *E. anophelis* C08 lysate, and nOMVs/iOMVs and incubated overnight at 35°C. The results indicated that only the total bacterial cell lysate showed a significant indentation of the inhibition zone (Fig. 7A and B), which is consistent with findings for the *bla*_CME_ and *bla*_GOB_ carbapenemase genes from the *E. anophelis* C08 genome (Fig. S3) (18). We further examined the cytotoxicity effect of OMVs and found that relative to the PBS control, iOMVs significantly reduced the A549 cell viability ratio (0.84) compared to that with nOMVs (0.99) (Fig. 7C). Furthermore, the opsonophagocytosis assay (OPKA) results suggested that iOMVs could provide a survival advantage to *E. anophelis* by protection from phagocytosis by macrophage cells. The killing percentages of nOMVs and iOMVs were −39.91% and −45.47% compared with the PBS control, respectively (Fig. 7D).

## DISCUSSION

Multidrug resistance in nosocomial bacterial strains is a frequent problem in clinics. *E. anophelis* is one of the leading causes of meningitis, septicemia, and urinary tract infection, more prevalent among neonates and children, and is associated with high mortality rates (19). In this study, the *E. anophelis* strain C08 was isolated from a 73-year-old patient. Traditional antimicrobial characterization was performed to reveal the multidrug resistance phenotype of the strain. MIC profiling provides insights into the multidrug resistance phenotype of *E. anophelis* strain C08, which is resistant to 13 individual antibiotics and 3 antibiotic-inhibitor combination drugs. Treatment with antibiotics inhibits cell wall biosynthesis and induces bubbling cell death, explosive cell lysis, and membrane vesicle formation, all of which have been observed in Gram-negative cells (9).

OMVs are spherical bodies enriched with toxins, virulence factors, and metabolically essential proteins that play an important role in cell-cell communication, bacterial pathogenesis, and bacterial survival (20). Nevertheless, the molecular mechanism underlying OMV production remains unclear. Our results suggest that OMVs could change the blebbing size and packaging caused by antibiotics, thereby prompting changes in the survival advantage as a response to specific environmental conditions. A similar mechanism has been reported in Pseudomonas aeruginosa and pathogenic Escherichia coli, in which polymyxin B induced OMV release based on membrane disruption (21). A detailed understanding of the proteome of OMVs cultured in an antibiotic stress environment will provide insights into the strategies that bacteria employ to promote their survival, including antimicrobial resistance. In this study, we cultured *E. anophelis* upon exposure to antibiotics and quantified the number of OMVs. Instead of spontaneous OMV blebbing, this coherent population of enriched and excluded OMVs and periplasmic components in OMVs suggests a specific vesicle biogenesis mechanism. Imipenem significantly increased OMV secretion, possibly since the disturbed cell wall structure or alteration of peptidoglycan dynamics is dependent on the quorum sensing diffusible signal system (22). The proteome annotation of membrane vesicles upon antibiotic stress was investigated, providing insight into their purpose and role in bacterial survival, defense, and resistance.

Proteins of the Rag/Sus family were the most highly expressed proteins in imipenem-treated cells. The Rag gene is a receptor antigen gene that encodes an outer membrane protein. RagA and -B are involved in the uptake of ligands, including carbohydrates, glycoproteins, and degraded proteins. A Rag mutant strain often shows reduced virulence, suggesting its role as a virulence determinant of the bacterium (23, 24). The presence of numerous proteins associated with carbohydrate metabolism, such as SusC/RagA, SucD/RagB, trehalase, the β-glucosidase BglX, glycoside hydrolase family 3 proteins, and β-glycosidase, highlights their potential roles in mosquitoes (25). Additionally, the expression of the molecular chaperonin GroEL denotes the stress condition of the bacterium, aiding in the prevention of protein misfolding, aggregation of proteins, and transport of peptides. The GroEL chaperonin or Hsp60 is a heat shock protein essential for the adequate folding of cellular proteins (26).

The essential proteins described below are transferases, a superfamily of enzymes involved in transferring functional groups that play crucial roles in various cellular processes and metabolic reactions (27). Imipenem-induced stress induced the expression of numerous essential survival proteins such as prenyltransferase, sulfurtransferase, acetyltransferase, aminotransferase, and nucleotidyltransferase. In addition, transferases, hydrolases, S9 and S46 family peptidases, and carboxypeptidases were also expressed. Peptidases hydrolyze peptide bonds, and proteolytic cleavage is essential for the bioactivity of numerous functional proteins (28). Enzymes such as amidases are highly expressed under antibiotic pressure, acting like autolytic enzymes (29), as observed in the present study with *N*-acetylmuramoyl-l-alanine amidase.

Any bacteria under stress will initiate tolerance and survival factors, such as the addiction module toxin RelE, referred to as toxin-antitoxin (TA) systems, indicating the survival need of the bacterial strain under antibiotic stress. Cells enter a quasidormant state, allowing them to survive under antibiotic stress, and the dormancy is reversed in the absence of the antibiotic. These proteins represent a good target for newer antibiotic designs promoting apoptosis and nonapoptotic cell death (30). The HmuY family protein, an essential heme binding protein in *E. anophelis*, is an outer membrane-associated lipoprotein and a major virulence determinant involved in the ability of bacteria to cause infection (31). Besides HmuY, ferrichrome-iron receptors and hemin receptors were also expressed, and the presence of hemolysins and siderophores in *E. anophelis* may play an important role in iron uptake and erythrocyte breakdown (25).

Numerous enzymes observed to be expressed in OMVs are involved in the pathogenesis and survival of the bacterial strain, and peptidylprolyl isomerase is one such protein. Peptidylprolyl isomerase is a chaperone involved in the structural modification of porin channels (32). The expression of metalloproteases such as M1, M13, and M48 also signifies a survival advantage. These metalloproteases are integral membrane proteins essential for peptide hydrolysis, the localization of proteins, and virulence (33). TonB-dependent receptors (TBDRs) are membrane transporters present in large numbers in *E. anophelis* and are involved in nutrient uptake (25). TBDRs are essential for the survival of and colonization by *E. anophelis*, and they exhibit different conserved domains associated with different substrates.

Likewise, PorT proteins (integral membrane protein-translocating porins), outer membrane proteins, and major facilitator superfamily (MFS) multidrug pump transporters were equally expressed. The expression of membrane proteins, porins, and efflux pumps greatly influences antibiotic resistance and, hence, the survival of the bacterium in the antibiotic-pressured environment. The presence of membrane proteins confers the intrinsic antibiotic resistance phenotype to *E. anophelis*. Numerous MFS transporters were observed to be expressed in OMVs, and these proteins play a role in antibiotic extrusion and facilitate the wide transport of substrates such as small solutes, sugars, and amino acids (34). These proteins are independently expressed in iOMVs and potentially contribute to the virulence and survival capabilities of *E. anophelis* (Fig. 7). Intriguingly, despite *E. anophelis* being resistant to imipenem, it differentially packages specific proteins in OMVs for reasons that remain to be determined. A recent study demonstrated that imipenem increases infection-related mortality by multidrug-resistant Klebsiella pneumoniae in a murine infection model (35). Meanwhile, here, imipenem increased the vesiculation and lethality of the carbapenem-resistant strain via mechanisms associated with bacterial membrane vesicles.

Cross talk between genes connects multiple pathways and their biological functions within cells (36). The MCODE clustering technique significantly reduced the chances of false-positive outcomes, improving the resilience and accuracy of the analyses (37). Functional and enrichment analyses of proteins identified in the OMVs from the control strain were performed, and proteins were classified into MCODE cluster 1 (TipA, LysM, TonB, OmpA, OMP_B-BRL, and uncharacterized proteins), cluster 2 (OMP_B-BRL, OmpA, heme receptor, lipocalin family protein, and uncharacterized proteins), and cluster 3 (DUF5074, cell surface protein, and YncE). The MCODE clusters of imipenem-treated OMV proteins were as follows: cluster 1 (GDSL-like lipase, endonuclease, carboxypeptidase family protein, and uncharacterized proteins), cluster 2 (TonB-dependent receptor, OmpA, and uncharacterized proteins), cluster 3 (TonB-dependent receptor, PKD domain-containing protein, and YncE family protein), and cluster 4 (SusE, SusB, TonB, SusD, RagB, and uncharacterized proteins). These clusters indicate that control and imipenem-induced OMVs were packed mostly with outer membrane proteins and glucosidase proteins.

The membrane barrel and periplasmic domains of the OmpA porin interact with peptidoglycans and are therefore responsible for the stability of the bacterial outer membrane. The β-barrel domain transfers some β-lactams into the bacterium, such as sulbactam and imipenem (38). Small hydrophobic compounds such as steroids, bilins, retinoids, and lipids are transported by lipocalins, a family of proteins expressed under stress conditions (39). YncE is involved in iron acquisition and is a transporter between the cytoplasmic and outer membranes. YncE is also crucial for the pathogenic mechanism of bacteria (40). The MCODE clustering method revealed that OMVs from both control and imipenem-treated cells were packed with outer membrane proteins and their closely associated proteins. Some stress-related proteins were also present, indicating that stress-related and outer membrane protein pathways were activated, and their proteins were packed into the imipenem-treated OMVs. Our proteome profiling results for *E. anophelis* indicate that OMVs can provide transfer factors concerning virulence, resistance, and cellular metabolism. Apart from these functional proteins, the presence of heme binding proteins, ferrichrome-iron receptors, hemin receptors, hemolysins, and siderophores emphasizes their role in the bacterium’s symbiotic relationship with mosquitoes.

In summary, the proteome of OMVs derived from the *E. anophelis* C08 strain was characterized *in vitro* under imipenem-induced-resistance conditions. Proteomic analysis showed that OMVs from imipenem-induced and control strains exhibited an enrichment of proteins/enzymes involved in antibiotic resistance, virulence, survival, stress, metabolism, and structure. In both control and induced cells, the outer membrane and extracellular proteins were packed into OMVs. OMVs produced in the presence and absence of imipenem contained several outer membrane and membrane transporter proteins. Most outer membrane proteins and transporters are involved in antibiotic resistance, transporting small molecules into/out of the cell. In particular, the MFS transporter and OmpA were identified in OMVs isolated from cultures grown in the presence of imipenem. Trehalase and PDZ domain-containing proteins were identified only in antibiotic-treated OMVs involved in host cell virulence and pathogenesis. Proteins such as short-chain dehydrogenase, MreC, and the ferrichrome-iron receptor, involved in biofilm biogenesis, were identified in OMVs derived from control and treated strains. Proteomic analysis of OMVs from both control and imipenem-treated strains showed that they were packed with essential stress, defense, and cellular metabolic proteins. Under antibiotic stress conditions, OMVs also contained several receptors and virulence- and toxin-related proteins that are strongly antigenic and promote the host immune response to *E. anophelis*. Using ClueGO analysis, a PPI network was constructed for proteins identified from imipenem-induced and control OMVs. Highly interactive protein clustering was identified using MCODE in Cytoscape. The study growth conditions with 1/2 the MIC of imipenem and the induced antibiotic resistance in the bacterium were clearly correlated with the PPI network interactions and the respective MCODE cluster information. In this study, OMVs have been shown to support the bacterial strain with more virulence and survival proteins and might represent the best source for developing a vaccine against emerging clinical pathogens, paving the way to finding the proteins essential for *E. anophelis* survival.

## MATERIALS AND METHODS

### Bacterial strain, growth conditions, and antimicrobial treatment.

The *E. anophelis* C08 strain was obtained from the Microbial Infections Reference Laboratory of the National Health Research Institutes, Taiwan. The clinical isolate was obtained from a patient with bacteremia and was cultivated aerobically in LB broth at 35°C with shaking at 250 rpm. A culture of *E. anophelis* C08 grown overnight (16 h) was inoculated into LB broth and incubated for 6 h with different antibiotics to reach the mid-log phase (optical density at 600 nm [OD_600_] = ~0.5). The bacterial culture grown overnight was mixed with 30% glycerol and stored at −80°C.

### Broth microdilution assay.

The broth microdilution assay was conducted according to Clinical and Laboratory Standards Institute (CLSI) guidelines (41). From an inoculum at a 0.5 McFarland standard, 100 μL was taken and diluted 1:100 in 10 mL cation-adjusted Mueller-Hinton broth (CA-MHB) (BD BBL; Thermo Fisher Scientific, Merelbeke, Belgium). The diluted inoculum (50 μL) was transferred to each well of a 96-well plate containing CA-MHB (50 μL) with or without antibiotics (1:2 dilution), resulting in an inoculum size of ~5 × 10^5^ CFU/mL. Finally, the 96-well plates were tightly sealed with adhesive foil and incubated for approximately 24 h at 35°C. Strains were designated susceptible (S), intermediate (I), and resistant (R) based on their respective MIC values and CLSI-defined interpretive criteria for non-*Enterobacteriaceae* organisms.

### Disk diffusion.

The Escherichia coli ATCC 25922 suspension (0.5 McFarland standard) prepared as described above was inoculated onto the entire surface of a Mueller-Hinton agar (MHA) plate using a sterile cotton-tipped swab. Four sterile paper disks (6 mm in diameter; BD Diagnostic Systems) were treated with imipenem (10 μg) plus 10 μL of an nOMV/iOMV solution (1 μg/μL), and imipenem (10 μg) and an *E. anophelis* C08 cell lysate solution (1 μg/μL) were placed onto the surface of each MHA plate. The plates were then incubated aerobically at 37°C for 24 h, and the diameter of the inhibition zone was then measured with a ruler.

### Isolation and purification of outer membrane vesicles.

Briefly, *E. anophelis* cultures were grown for 6 h in the required quantity of LB broth with and without the sub-MIC of imipenem until the log phase for OMV isolation (OD_600_ = ~0.5). The culture was pelleted by centrifugation at 6,000 × *g* at 4°C for 20 min, and the supernatant was filtered using 0.22-μm polyether sulfone (PES) membrane filters (Express Plus; Millipore, Darmstadt, Germany). The filtrate was concentrated in continuous diafiltration mode using a Minimate tangential flow filtration (TFF) system and a capsule with an Omega 100,000-molecular-weight (100K) membrane (Pall Corporation, Ann Arbor, MI, USA). OMVs were isolated from 10 mL of the concentrated supernatants using a qEV10 size exclusion column (Izon, New Zealand) with degassed 1× PBS. The flowthrough fractions were subjected to ultracentrifugation at 150,000 × *g* for 3 h at 4°C to pellet the OMVs. The OMVs were then washed in PBS, followed by ultracentrifugation at 150,000 × *g* for 3 h at 4°C. The purified OMV pellets were carefully and gently resuspended in 1,000 μL PBS containing a protease inhibitor cocktail (Thermo Fisher), and the protein patterns were determined using SurePAGE 4-to-20% Bis-Tris gradient gels (GenScript, Piscataway, NJ, USA). The vesicle suspension was checked for bacterial contamination by plating onto LB agar plates, and pure suspensions were stored at 4°C for use within 2 weeks or at −80°C for future analysis.

### TEM imaging.

The OMVs isolated from cultures grown with and without ampicillin, amikacin, chloramphenicol, colistin, meropenem, minocycline, imipenem, polymyxin B, and tigecycline were fixed in 2% paraformaldehyde and 2.5% glutaraldehyde in PBS and thoroughly mixed for TEM analysis. Fixed samples were washed in 0.1 M sodium cacodylate (pH 7.24), postfixed with buffered 2% OsO_4_, washed with water, and dehydrated in a graded ethanol series (25 to 100%) with 25% increments. The samples were filtered in a graded series of Epon resin (Ted Pella Inc., Redding, CA, USA) for 2 days, embedded in fresh Epon resin, and polymerized at 60°C for 48 h. Purified OMV pellets were resuspended in 2% paraformaldehyde and allowed to free-float onto a 200-mesh carbon-coated Formvar nickel grid (Electron Microscopy Sciences, Hsin An Instruments Co. Ltd., Taiwan) for 5 min. The excess solution was dried off with filter paper, and the sample grid was then floated on a 10-μL droplet of 1% aqueous uranyl acetate for 30 s. The stain was removed with filter paper, and the sample was air dried. Finally, the samples were imaged using a Hitachi (Tokyo, Japan) HT7700 transmission electron microscope.

### Zeta potential of OMVs.

Zeta potential analysis was conducted according to Izon’s instructions for V3.1 charge analysis using NP100 and CPC100 particles. The calibration particles were measured at three applied voltages; particles at the highest voltage were measured at two external pressures. Data obtained from measured samples and calibration particles were evaluated using zeta template V3.1a provided by Izon.

### Nanoparticle tracking analysis.

The size distributions of the nanoparticles were determined using nanoparticle tracking analysis (NTA) (NanoSight NS300; Malvern Panalytical, Malvern, United Kingdom). Samples were diluted 100 times in a total volume of 1 mL of PBS. Diluted OMVs were measured based on Brownian movement and loaded onto a NanoSight NS300 system, and the particle size was recorded for 60 s per technical replicate. Five technical replicates and three biological replicates were analyzed per sample. The NTA determined the particle number per milliliter of OMV solution. The protein concentration in each sample was quantified using the bicinchoninic acid (BCA) assay (Thermo Fisher, Waltham, MA, USA).

### OMV-associated proteins identified by mass spectrometry.

The OMV solution was extracted using SDS-PAGE sample buffer at 100°C for 5 min, and the proteins were isolated using one-dimensional gel electrophoresis (1 cm). The excised gel was first destained and then reduced with 10 mM dithiothreitol (DTT; Merck, Darmstadt, Germany) at 60°C for 45 min, followed by cysteine blocking with 55 mM iodoacetamide (IAM; Sigma-Aldrich, St. Louis, MO, USA) at 25°C for 30 min. Samples were digested with sequencing-grade modified porcine trypsin (Promega, Madison, WI, USA) at 37°C for 16 h. The peptides were then extracted from the gel, dried by vacuum centrifugation, and reconstituted with 0.5% formic acid before analysis. The digested peptides were diluted in high-performance liquid chromatography (HPLC) buffer A (0.1% formic acid) and loaded onto a reverse-phase column (Zorbax 300SB-C_18_, 0.3 by 5 mm; Agilent Technologies, Santa Clara, CA, USA). The liquid chromatography (LC) apparatus was coupled to a two-dimensional (2D) linear ion trap mass spectrometer (Orbitrap Elite ETD [electron transfer dissociation]; Thermo Fisher) operated using Xcalibur 2.2 software (Thermo Fisher). The 20 data-dependent tandem mass spectrometry (MS/MS) scan events were followed by one MS scan for the 20 most abundant precursor ions from the preview MS scan. The *m/z* values selected for MS/MS were dynamically excluded for 80 s, with a relative mass window of 15 ppm.

### Bioinformatic classification of the identified proteins.

Data analysis was carried out using Proteome Discover software (v1.4; Thermo Fisher Scientific), including the reporter ion quantifier node for tandem mass tag (TMT) quantification. *E. anophelis* protein sequences were obtained from the UniProt database (http://www.uniprot.org/). Protein localization was predicted using Cello2go (http://cello.life.nctu.edu.tw/cello2go/) (42). The functions of OMV proteins were searched by BLAST analysis against the Clusters of Orthologous Groups of proteins (COG) database (43).

### PPI network analysis.

PPI data were downloaded using a String database (44). A PPI network of OMV proteins was constructed, analyzed, and visualized using Cytoscape 3.9.0 software (45). Using ClueGO v2.5.8 and CluePedia v1.5.8 in Cytoscape, Gene Ontology enrichment analysis was performed for OMV proteins (46). Using the MCODE application in Cytoscape, the highly interconnected regions in the PPI network were identified using the following parameters: a node score cutoff of 0.2, a K-core value of 2, and a degree cutoff of 2 (17).

### Cytotoxicity assay.

A549 cells were seeded into 96-well plates (5,000 cells per well) with 10 μg nOMVs/iOMVs and incubated for 4 and 24 h, after which cell viability was measured and quantified using cell counting kit 8 (CCK-8) (TEN-CCK8; Tools) according to the manufacturer’s instructions using a microplate reader (OD_450_). In each experiment, the relative cytotoxicity ratio was calculated as the OD value of OMVs relative to that of PBS buffer (negative control).

### Opsonophagocytosis assay.

A phagocytosis assay was conducted as previously described (47). J774A.1 murine macrophages (ATCC CCL-240) were cultured in Dulbecco’s modified Eagle’s medium (DMEM; GeneDireX) supplemented with 10% heat-inactivated fetal bovine serum (Gibco) and 100 U/mL penicillin-streptomycin (GeneDireX), and cells were incubated at 37°C with 5% CO_2_. J774A.1 cells were stimulated with 100 nM phorbol myristate acetate (PMA; Sigma-Aldrich) for 3 days. The bacterial pellet was washed twice with PBS, resuspended in DMEM, and used in the assays. Reactions were initiated by incubating 2 × 10^5^ CFU (multiplicity of infection [MOI] = 2) of *E. anophelis* C08 with heat-inactivated (56°C for 30 min) test serum and nOMVs/iOMVs in the wells. After a 2-h incubation with gentle shaking (80 rpm), the samples were serially diluted and plated. Bacterial killing rates were determined by comparing the number of reduced CFU with those observed using PBS-immunized antiserum. Hyperimmune serum from OMV-immunized mice was used as a positive control (data not shown, animal studies were approved by the National Defense Medical Center Institutional Animal Care and Use Committee, NDMC IACUC-19-250).

### Statistical analysis.

All assays were performed in triplicate, and the standard deviations (SD) were calculated. GraphPad Prism 9.0 (GraphPad, San Diego, CA, USA) was used to perform the statistical analyses. Statistical significance was set at a *P* value of <0.05. One-way analysis of variance (ANOVA) with Bonferroni’s multiple-comparison *post hoc* test was used to compare multiple groups.

### Data availability.

The mass spectrometry proteomics data obtained from biological replicates have been deposited in the ProteomeXchange Consortium via the PRIDE (48) partner repository with the data set identifiers PXD033416 and PXD033858.
