# Supplementary figures and images for: Proteomic Network of Antibiotic-Induced Outer Membrane Vesicles Released by Extensively Drug-Resistant Elizabethkingia anophelis
Source: Microbiol Spectr. 2022 Jul 19;10(4):e00262-22. doi: 10.1128/spectrum.00262-22 (PMC9431301; doi:10.1128/spectrum.00262-22)

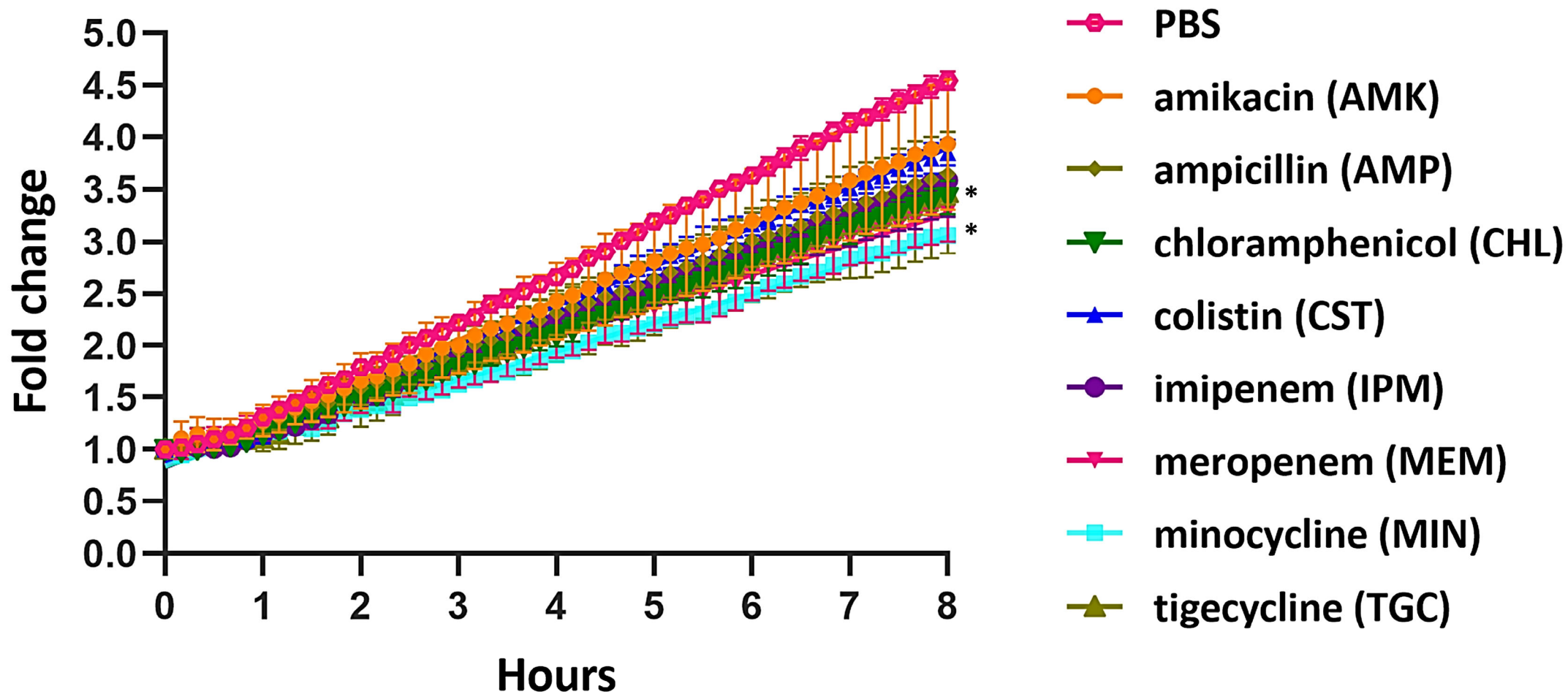

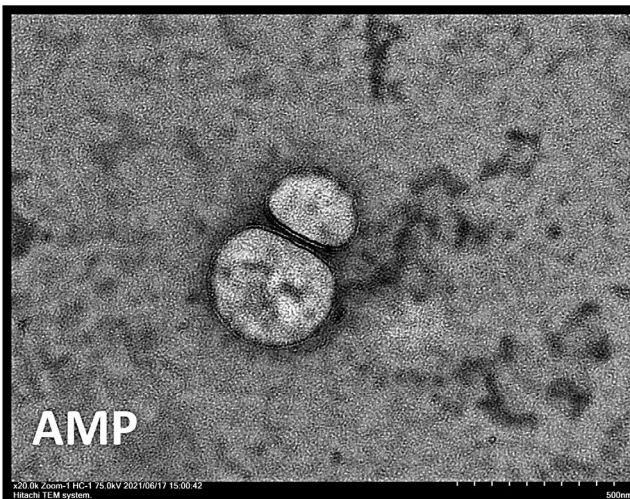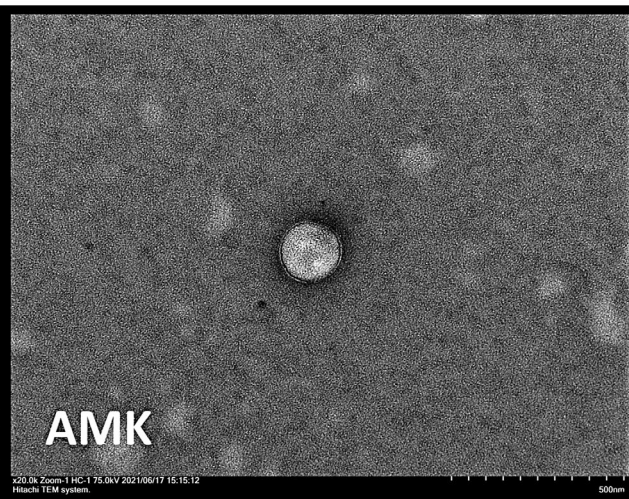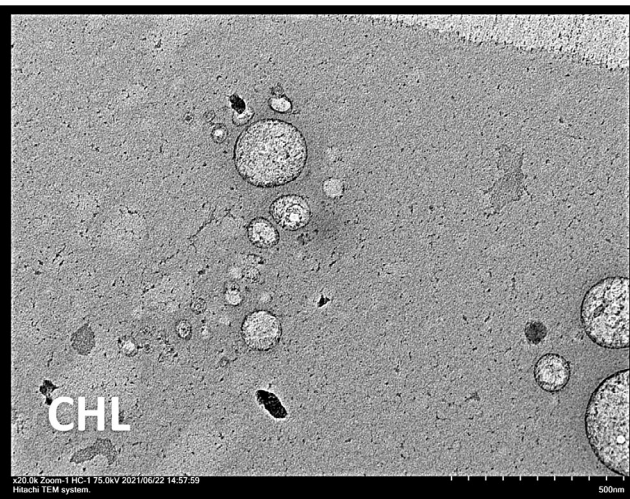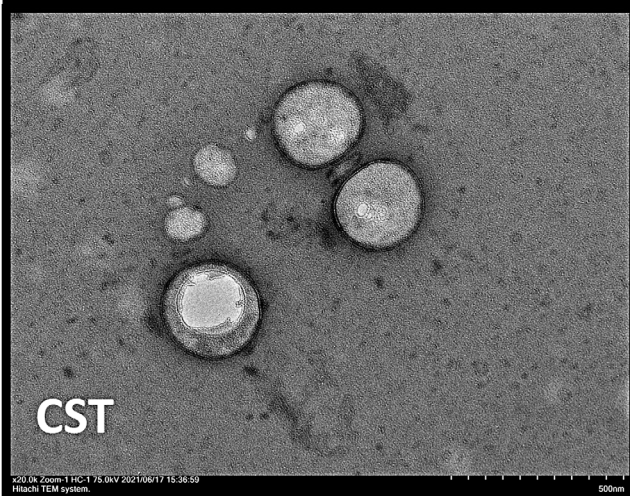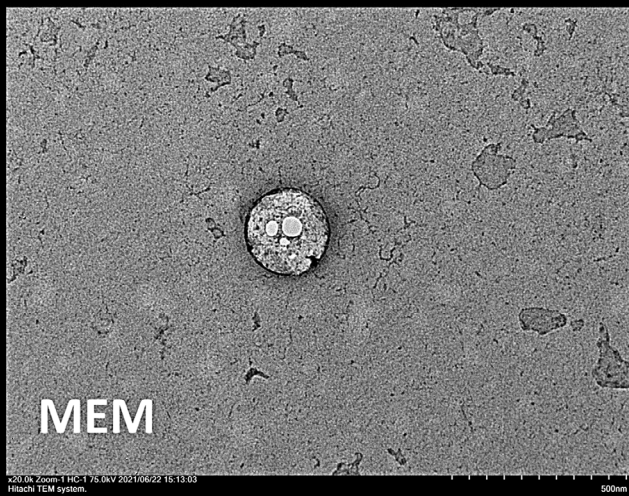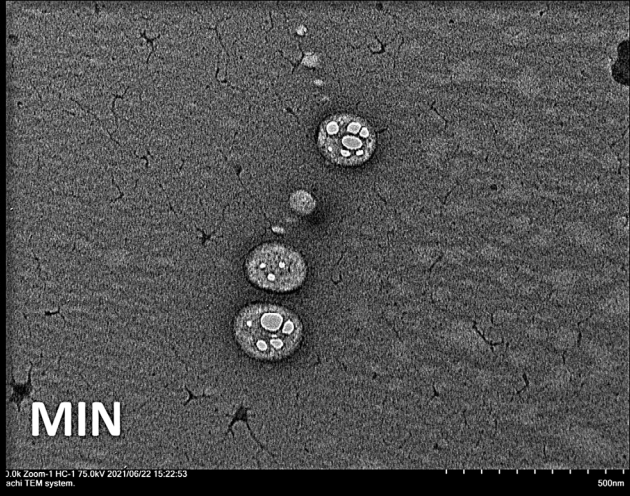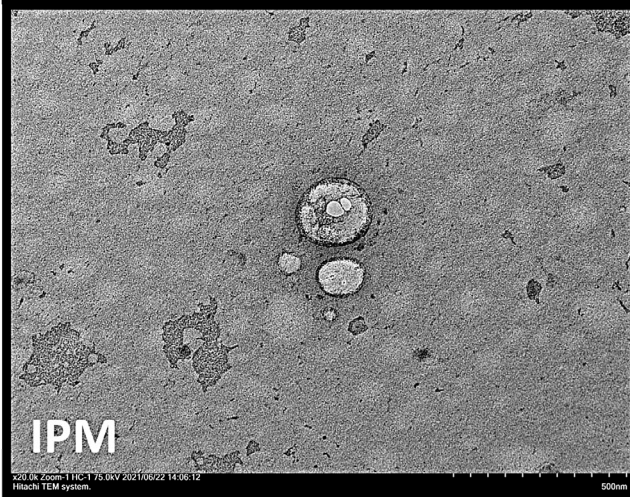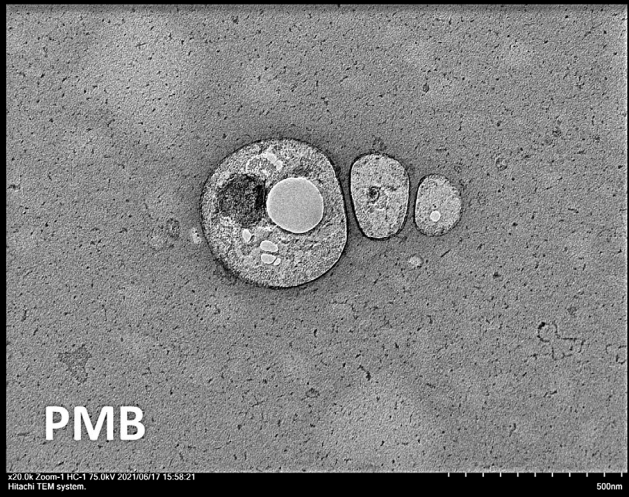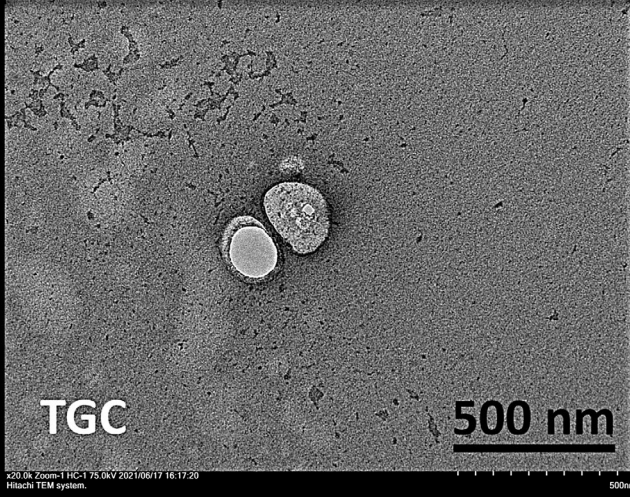

500 nm

**M    1    2    3    4    5    6    7    8    9    10    11    12    13    14    M**

**1500 bp** 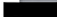  
**1000 bp** 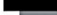  
**500 bp** 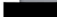

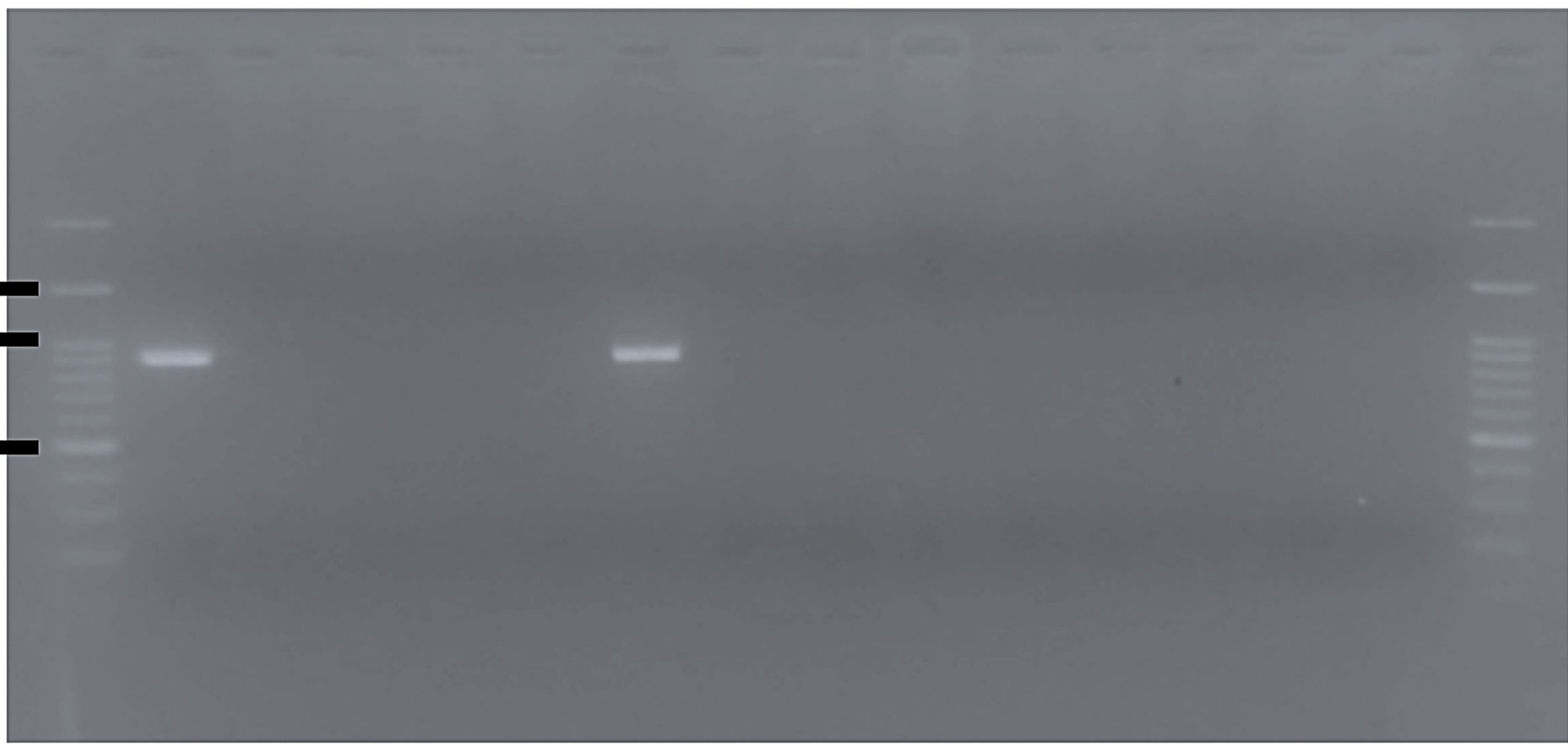

Supplement: Supplemental file 1 — Supplemental material. Download spectrum.00262-22-s0001.pdf, PDF file, 3.6 MB [file spectrum.00262-22-s0001.pdf]
